# Dysregulation in the microbiota by HBV and HCV infection induces an altered cytokine profile in the pathobiome of infection

**DOI:** 10.1016/j.bjid.2024.104468

**Published:** 2024-11-28

**Authors:** Marcos Daniel Mendes Padilha, Francisco Tiago de Vasconcelos Melo, Rogério Valois Laurentino, Andrea Nazaré Monteiro Rangel da Silva, Rosimar Neris Martins Feitosa

**Affiliations:** a Universidade Federal do Pará (UFPA), Instituto de Ciências Biológicas, Laboratório de Virologia, Belém, PA, Brazil; b Universidade Federal do Pará (UFPA), Instituto de Ciências da Saúde, Health Sciences, Belém, PA, Brazil

**Keywords:** Dysregulated microbiota in HBV infection, Microbiome and viruses, HBV and HCV co-infection, Viral hepatitis

## Abstract

Viral hepatitis is a public health problem, about 1 million people die due to complications of this viral disease, the etiological agents responsible for inducing cirrhosis and cellular hepatocarcinoma are HBV and HCV, both hepatotropic viruses that cause asymptomatic infection in most cases. The regulation of the microbiota performs many physiological functions, which can induce normal intestinal function and produce essential nutrients for the human body. Metabolites derived from gut microbiota or direct regulation of host immunity and metabolism have been reported to profoundly affect tumorigenesis in liver disease. If the microbiota is unbalanced, both exogenous and symbiotic microorganisms can affect a pathological process. It is well understood that the microbiota plays a role in viral diseases and infections, specifically the hepatic portal pathway has been linked to the gut-liver axis. In HBV and HCV infections, the altered bacterial representatives undergo a state of dysbiosis, with subsequent establishment of the pathobiome with overexpression of taxons such as *Bacteroides, Clostridium, Lactobacillus, Enterobacter,* and *Enterococcus*. This dysregulated microbiome induces a microenvironment conducive to the development of hepatic complications in patients with acute and chronic HBV and HCV infection, with subsequent dysregulation of cytokines IFN-α/β, TNF-α, IL-1β, TGF-β, IL-6 and IL-10, which alter the dysfunction and damage of the hepatic portal system. In view of the above, this review aimed to correlate the pathophysiological mechanisms in HBV and HCV infection, the dysregulation of the microbiome in patients infected with HBV and HCV, the most altered cytokines in the microbiome, and the most altered bacterial representatives in the pathobiome of infection.

## Introduction

The microbiome is composed of trillions of microorganisms, including bacteria, fungi, and protists, among which bacteria are the most predominant.^1^ Changes in the gut microbiota influence the gut and the liver, which are closely connected by the so called gut-liver axis.^2^ Hepatocellular Carcinoma (HC) is an aggressive cancer and develops almost exclusively in patients with chronic liver disease and cirrhosis. Although chronic liver disease is multifactorial, it's primarily related to infections by Hepatitis B (HBV) and Hepatitis C (HCV) viruses, nonetheless, there is growing evidence supporting a determinant role of the bacterial microbiome in the development of chronic liver disease.^1^

The microbiota is an important determinant of the symbiotic relationship in the onset and in the clinical evolution of liver diseases. Gut dysbiosis is associated with viral hepatitis, mainly by HBV and HCV induced infection. Metabolites derived from gut bacteria and cellular components are vital molecules that affect liver function and modulate pathology in viral hepatitis.^2^

Regulation in the microbiota performs many physiological functions, which can induce normal bowel function and produce nutrients such as vitamin K, B12, short-chain fatty acids, and essential amino acids for the human body. Metabolites derived from gut microbiota or direct regulation of host immunity and metabolism have been reported to affect tumorigenesis remarkably. The viral infection reduces the number of beneficial bacteria and increases the number of harmful bacteria in the body; if the microbiota is unbalanced, both exogenous and symbiotic microorganisms can affect a pathological process in the individual.^3^

Cytokines are potent cell growth regulators with immunomodulatory and antiviral activity of innate and adaptive immunity. Most cell types produce type I Interferon (IFN-α/β), while IFN-γ is produced only by immune cells, including Natural Killer (NK), TCD4^+,^ and TCD8^+^ lymphocytes. IFNs trigger complex signaling, resulting in the transcription of hundreds of IFN-Stimulated Genes (ISGs). The levels of these cytokines increase in viral infections, and the sequence of events or types of IFNs directly affects the pathobiome of infection.^4^

Various microorganisms influence viral infections in the surrounding environment in the target tissue. Thus, the correlation between the type and balance of the commensal microbiota is crucial in establishing infection and pathogenicity. It is evident that the microbiota profile under normal conditions influences the progression of viral diseases. It is crucial to elucidate the interactions between viruses, target tissue, and the surrounding microbiome, which must have different relationships with each virus to understand the pathogenesis underlying viral infections.^5^

The microbiota represents the totality of microbes associated with the human organism, while the microbiome consists of all microbes and their genes. The central part colonized by microbes is the gastrointestinal tract, other parts like skin or airways are also colonized, but in a lesser extent. The microbiota continually changes, in recent years, there have been advances in the understanding of the microbiome and its interaction with the host, and the gut-liver axis is part of these discoveries integrating the modifications of the microbiome and dysbiosis in the liver pathologies.^6^

Thus, this review sought to correlate the immunopathological mechanisms related to the microbiome, the pathophysiology of HBV and HCV related to microbiome alteration, the dysregulated cytokine profile in the microbiome of hepatocarcinogenesis, and which taxons and cytokines are most altered in the pathobiome of HBV and HCV infection.

### Viral hepatitis

Hepatitis caused by viruses is still a public health challenge, sparking epidemics dating back to ancient times, with outbreaks documented 5000 years ago in China and similar descriptions by Hippocrates in the fifth century B.C. on Thassos Island.^7^ The main factors for the development of hepatitis include HBV, HCV, alcohol abuse, and non-fatty liver disease.^8^ The mechanisms of carcinogenicity are hypothesized to be multifactorial, including disease duration, viral load, and ethnicity.^7^

This viral disease is responsible for 80% of HC cases worldwide, HC patients are usually asymptomatic at the early stage and often have typical symptoms when they are at an advanced stage, such as liver pain, jaundice, ascites, and liver failure. Chronic HBV infection affects approximately 250 million people, of whom between 48 and 60 million are co-infected with Hepatitis D Virus (HDV), and 2.6 million are co-infected with HCV. Individuals co-infected with HBV and HCV have a higher incidence of HC and a worse prognosis compared to monoinfection.^8^

About 1 million people die due to sequelae resulting from chronic HBV or HCV infection related to end-stage liver disease and HC. Liver injury is known to be caused by the immune system, with specific TCD8^+^ lymphocytes being the main effectors, where their depletion delays the clearance of HBV and HCV in acute disease.^9^

HBV and HCV are enveloped viruses with multiple genotypes or different replication strategies; both have developed mechanisms to establish chronic hepatitis.^9^ HBV is differentiated into 10 known genotypes (A-J), patients infected with genotype C have higher levels of viral replication, ALT (Alanine Transaminase), IFNs and worse clinical response. The possible relationship between B/C genotypes and T-helper cells was investigated, and it was observed that high proportions of HBV DNA in patients with genotype C may be associated with lower levels of T-helper cells in peripheral blood, causing low levels of IL-21. It was also described that patients with genotype C more frequently develop chronic infections, in addition, the rate of HBeAg clearance is lower in individuals with genotypes C and D.^10^

Six genotypes (1‒6) of HCV were identified worldwide, each comprising several subtypes, it was observed that genotype 1b appears to be more prevalent in patients with liver cirrhosis, with a rate of evolution to chronicity being reported after acute exposure of the genotype 1b of 92%. It has been suggested that patients with genotype 1b have a lower response to treatment than those with genotypes 2 and 3. Genotypes that undergo mutations in amino acid sequences have already demonstrated immune escape and low production of cytokines by lymphocytes.^11^

Certain genotypes of HBV or HCV and their subtypes can influence the activity of T, B, NK cells and lead to cytokine dysregulation, this imbalance in immune homeostasis, leads to changes in the balance of the microbiota, such as the production of IL-1β, IL-18 and IL-22 that are regulated by the microbiota to facilitate the development of the adaptive immune response against viral infection. The transition from the microbiome to the pathobiome in hepatitis B and C, with the interruption of mutually beneficial interactions of the microbiota, may direct mechanisms susceptible to more aggressive cases of cirrhosis and HC through the intestine-liver axis.^12^

### Microbiota and hepatocellular carcinoma

**Fig. 1:**
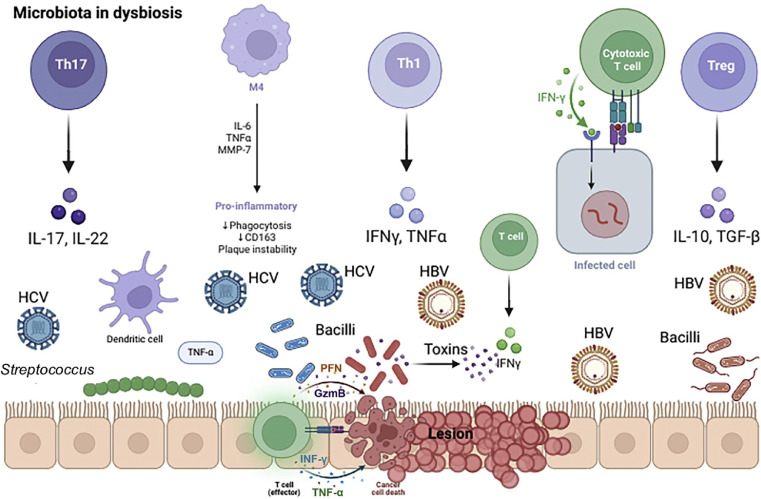
Microbiota in dysbiosis.

The microbiota plays an essential role in the maturation of the immune system. The human microbiome of an adult tends to consist of approximately 90% Bacteroidetes and less than 1% *Enterobacter* and *Enterococcus*. The gut-liver axis involves multiple inflammatory processes, cells, cytokines and many probiotics have been shown to reduce the impact of various forms of acute liver injury. The microbiota (Fig. 1) plays a role in the initial manifestation of hepatic encephalopathy, bacterial peritonitis, and systemic sepsis, and it is suggestive that the mechanisms by which HBV promotes pathogenesis are mediated in part by the microbiota.^13^

Any change in cross-response can lead to a state that affects bowel and liver function. HCV infection is associated with gut microbiota dysbiosis, which can initiate a cycle of inflammation and metabolic disorders contributing to pathogenesis. In the mouth and pharyngeal region, the microbiota is mainly composed of commensal bacteria of the genus *Moraxella, Haemophilus, Neisseria* and *Streptococcus*. Many microorganisms are symbiotically occupying the intestine, in relation to bacteria the most predominant phyla are Bacteroidetes, Firmicutes, Actinobacteria and Proteobacteria.^14^

Translocation of microbial products in the gastrointestinal tract, bacterial peptidoglycan, flagellin, and metabolic by-products may aggravate the clinical course of patients with chronic liver disease, liver dysfunction, or cirrhosis. Virulence factors such as fimbria, afimbrial adhesins, toxins from gut bacteria such as *Escherichia coli, Clostridium difficile, Bacteroides fragilis* and *Clostridium perfringens*, may provide mechanisms for these bacteria to disrupt the balance by inducing virulence genes.^15^

Microbiota products (Fig. 1) activate the innate immune system to drive pro-inflammatory gene expression, being an intricate component of the receptor system that recognizes conserved features of bacterial products such as peptidoglycan, lipopolysaccharide, flagellin, and bacterial DNA. It also has a variety of effects on metabolic, growth and differentiation pathways in the host phenotype. While the immune system plays an important role in containing pathogens, it can also induce liver disease mediated by dysregulation of immune cells and cytokines.^16^

Inflammatory bowel disease is associated with risk variants in the human genome and dysbiosis in the microbiome, with several susceptibility genes linked to autophagy regulation such as *ATG16L1* or microbial sensors that activate autophagy such as *NOD2*. The commensal *B. fragilis*, delivers immunomodulatory molecules to immune cells through outer membrane vesicles, *ATG16L1*-deficient dendritic cells do not induce regulatory T-cells (Treg) to suppress mucosal inflammation causing inflammation.^17^

Alteration in gut homeostasis alters the microbiome contributing to the pathogenesis of many disorders, including liver disease. A common feature of cirrhosis is an increase in potentially pathogenic bacteria, accompanied by a reduction in beneficial bacteria. Most of these patients with cirrhosis have bacterial overgrowth, increased endotoxin levels, systemic inflammation, and production of ammonia (a bacterial byproduct), with peritonitis related to migration of bacteria to the peritoneal cavity or circulation in cases of end-stage liver disease.^18^

The translocation of the bacterial components called Pathogen-Associated Molecular Patterns (PAMPs), triggers inflammatory responses through the Toll-Like Receptor (TLR), followed by the activation of NF-kB, whose overexpression induced by lymphocyte lymphotoxins, by the NF-kB, TNF-α and IFNs signaling genes, acts as a promoter of hepatocarcinogenesis, being a crucial determinant in tumor recurrence. The translocation of bacteria and bacterial PAMPs is common in chronic liver disease, unlike the intestine, the liver is not in direct contact with the bacteria, however it is the first target of the bacteria through the hepatic portal circulation, favoring cellular hepatocarcinoma.^19^

### Physiopathogenesis of HBV infection

The acute phase of hepatitis B is characterized by myalgia, nausea, vomiting, fatigue, malaise, change in the sense of smell or taste, abdominal pain, runny nose, photophobia, headache, and diarrhea. Acute liver injury is confirmed by an increase in serum alanine transaminase activity. The activities of alkaline phosphatase and γ-glutamyltransferase may also be increased in patients with acute liver injury, but their activities are generally lower than those of alanine transaminase.^20^

The immune response, apoptosis, liver inflammation and liver fibrosis, create an ideal substrate for the onset of HC. Mutations in the PreS1/S2 domain of HBV cause an accumulation of large, altered surface proteins, resulting in oxidative stress, integration of the HBV genome into the host, genomic instability, or direct involvement of cancer-associated genes. The Hbx protein of the virus may have a pro-carcinogenic effect because it causes the production of reactive oxygen species, which can interfere with the Jak/STAT, RAS/RAF/MAPK or Wnt/β catenin pathways and inactivation of *p53* causing epigenetic modifications in the DNA.^21^

Chronic infection has three phases, the immunotolerant, immunoactive, and low or non-replicative phase. In immunotolerant, HBsAg and HBeA antigens are detectable, serum HBV DNA levels are elevated, and serum aminotransferases are normal or minimally elevated. In immunoactive patients, serum HBV DNA levels decrease, and aminotransferase levels increase, aminotransferase seizures may be observed in some patients, followed by anti-HBe seroconversion of HBeAg. In the persistence replication phase, viral replication continues, but at a very low level, suppressed by the host's immune response.^22^

Chronic HBV infection is characterized by an expansion of TCD4 and TCD8 lymphocytes. A comprehensive analysis of TCD4^+^ and CD25^+^ (Tregs) cells in the blood at different stages of HBV infection revealed that patients with severe chronic hepatitis B have a significantly higher number of Tregs compared to acute infection, and that one of the reasons for the hyporesponsiveness of T-cells is due to the mass production of Treg. Chronic infection is associated with impaired proliferation, cytokine production, and cytotoxic functions of HBV-specific effector T-cells.^23^

HBV has different associated mechanisms to promote tumorigenesis, specifically through the activation and deactivation of various pathways causing HC. In addition, HBV has the unique characteristic among hepatotropic viruses of generating HC in the absence of cirrhosis, although most cases occur in cirrhotic patients. One mechanism identified is that of mutant genes, such as *PYCR2* (a crucial component in proline biosynthesis responsible for collagen), *ADH1A* (enzyme that metabolizes a variety of compounds contributing to fat accumulation in the liver), *TERT* (its resulting protein is a subunit of telomerase, being dysregulated and destabilizing genomic integrity), and *RPS6KA3* which is often maintained in somatic mutations by dysregulating Toll-like signaling, associated with HBV.^24^

Populations of Antigen-Presenting Cells (APCs) are inefficient in presentation to effector T-cells, with a propensity to induce tolerance of T-lymphocytes instead of activation, due to poor expression of co-stimulatory molecules, upregulation of PD-1 and PD-L2 ligands after IFN stimulation and production of immunoregulatory cytokines such as IL-10 and TGF-β.^25^

### Physiopathogenesis of HCV infection

Most patients with newly acquired HCV have no symptoms of infection, symptomatic patients may present with jaundice and elevated alanine aminotransferase levels relative to the normal limit. Viral replication is extremely robust, producing about 10 trillion viral particles per day, its ability to antagonize the antiviral response is crucial for viral persistence. Several non-structural HCV proteins, such as E2,3/4A, have been implicated in the inhibition of IFN-inducible genes or key components of the IFN signaling pathway through multiple mechanisms.^26^

The hepatotropism and lymphotropism of this virus have been clearly demonstrated in the infection of lymphoid tissue and may represent a decisive factor for lymphoproliferative autoimmune diseases caused by viruses. Some authors have suggested a pathogenetic role in cryoglobulinemia, a systemic disease sustained by expansion of B-lymphocytes. HCV infection is also often associated with the circulation of autoantibodies or mixed cryoglobulinemia, this infection presents a translocation in which it can activate the *Bcl-2* proto-oncogene.^27^

Liver damage induces a persistent cycle of necroinflammation and hepatocyte regeneration, resulting in mutations in hepatocytes and expansion of degenerated cells, leading to the development of HC.^28^ HCV infection induces TGF-β1 through the production of reactive oxygen species, p38 pathways MAPK, JNK, ERK, and NF-kB. PDGF platelet-derived growth is the most potent mitogenic signal, along with integrins, pro-inflammatory JNK activation by the cytokine IL-1β can alter TGF-β signaling from tumor suppression to oncogenesis.^29^

Within the infected liver, double-stranded viral RNA replication intermediates are detected by Pathogen-Associated Molecular Pattern Receptors (PAMPs), resulting in the activation of transcription factors IRF3/7 and NF-kB with induction of ISGs. HCV antagonizes these antiviral responses, the responses of TCD8^+^ lymphocytes during infection, although critical for viral clearance, are able to minimally eliminate HCV due to its persistence in the cell. HCV also impairs a tumor suppressor substrate histone H2AX, when phosphorylated (γ-H2Ax) acts as a DNA repair factor recruitment platform to sites of damage, inhibition of γ-H2Ax by HCV can disrupt repair, contributing to hepatocarcinogenesis.^30^

HCV infection is associated with higher expression levels of IL-6, IL-8, IL-12, TNF-α, and macrophage inflammatory protein 1b. It has been suggested that immunity be skewed towards Th2 response type associated with HCV persistence. Patients with HC have higher levels of IL-10 in their peripheral blood mononuclear cells compared to individuals with self-limiting hepatitis. High expression of IL-10 was accompanied by low or undetectable serum levels of IL-12 and IFN-γ, which are involved in T-lymphocyte-mediated immunity and viral clearance.^31^

### Immune system and microbiota

The gut immune system monitors microbial communities flowing from the lumen and under healthy conditions, reacts against potentially pathogenic organisms by inducing inflammation, while maintaining tolerance to most members of the commensal microbiota. The ability to discriminate pathogens is mediated by PRRs including TLR families, Nucleotide-binding Oligomerization Domain-Like Receptors (NOD-NLRs), C-type Lectin Receptors (CLRs), Cytosolic DNA Receptors (CDRs), and RIG-I Receptors (RLRs).^32^

TLR2 is capable of triggering the proliferation and production of cytokines in particular IL-2, IFN-γ of effector T-cells activated via TCR, and in TCD8^+^ cells TLR2 induces T-bet, IFN-γ and TNF-α activity. The activation of TLR5 signaling has a pro-inflammatory effect by regulating the production of IL-17 and IL-22 which, in turn, promote antimicrobial defense for pathogen elimination. TLRs 7/8 mount a response in the form of IFN and ISGs, whereas TLR9 comes into contact with unmethylated DNA, increases the expression of type I IFN, halting viral replication, and initiating the destruction of infected cells.^32^^,^^33^

The active receptor for lipopolysaccharides is the CD14/TLR4/MD2 complex which secretes many pro-inflammatory cytokines (Table 1), including TNF-α, IL-1, IL-6, and chemokines. The mixture of various probiotics and *Bifidobacterium* with galactooligosaccharides and fructo-oligosaccharides has a defensive effect against infections, aggregating the production of TNF-α, IL-4, IFN-γ and TLR2 expression. In most liver diseases, especially cirrhosis, dysbiosis in the gut increases with the microbial representatives Proteobacteria, *Enterobacteriaceae* and *Veillonellaceae*, while the taxons Bacteroidetes and *Lachnospiraceae* decrease.^34^

**Table 1:** Altered biomarkers in the microbiota of HBV and HCV infected patients.

**Effect of altered microbiota in patients with viral hepatitis**				
**HBV infection**				
**Altered microbiome**	**Biomarkers**	**Immunological outcome**	**Clinical aspects**	**References**
↑*Bacteroides*, ↑Actinobacteria,	↑IL-10, ↑IL-13, ↑TGF-β	↓Inhibition of the immune response	↑Hepatocellular carcinoma	^1^^,^^45^
↑*Alloprevotella*	↑IL-1β, ↑IL-18	↑Activation and production of active caspase 1	↑Hepatocellular carcinoma	^4^^,^^46^
↑*Pseudobutyrivibrio*, ↑*Lachnoclostridium*	↓TNF-α, ↑IFN-γ, ↑IL-6	↑T-cell lymphotoxins	↑Hepatocellular carcinoma	^3^^,^^45^
↑*Phascolarctobacterium*	↑TNF-α, ↓IFN-β	↑TCD8^+^ lymphocytes, ↓Immune inhibitor	↑Chronic infection, ↓Hepatic fibrosis	^2^^,^^41^
↓*Verrucomicrobia*	↓IFN-γ	↓NK response	↑Chronic infection	^1^^,^^48^
↑*Ruminoclostridum*, ↓*Escherichia*, ↓ *Enterococcus*	↑IL-35	↓STAT 1 activation	↑Induction of hepatocarcinogenesis	^3^^,^^42^
↑*Clostridium sensu stricto*, ↑*Actinomyces*,	↓IL-33	↓Cytokine production	↑Persistent infection	^52^^,^^42^
↑*Enterobacteriaceae*	↑IL-8	↑ERK, ↑JNK, ↑CREB e ↑C/EBP	↑Exacerbation of liver injury	^14^^,^^46^
↑Gammaproteobacteria, ↓*Bifidobacterium*	↑IL-23	↑ERK/JNK	↑Disease progression	^34^^,^^42^
↑*Alcaligenaceae*, ↑*Porphyromonadaceae*	↑IL-29	↑IL-8	↑Persistent infection	^34^^,^^46^
↑*Faecalibacterium prausnitzii*	↓IL-12	↓NK response	↑Disease progression	^3^^,^^48^
↓Proteobacteria	↑IL-22	↑Th17	↑Cirrhosis of the liver	^33^^,^^49^
↑*E. coli*, ↑*Prevotella*	↑IL-17	↑↓IL-22	↑Hepatic fibrosis	^35^^,^^49^
↑*Clostridium difficile*	↑CXCL-9	↑TCD8^+^ lymphocytes	↑Hepatocellular carcinoma	^77^^,^^50^
↓*Asaccharobacter*, ↓*Parabacteroides*	↑CXCL-10	↑NK, ↑Th1	↑ALT	^57^^,^^50^
↑*Porphyromonadaceae*	↓IFN-α, ↓Osteopontin (OPN)	↓Hepatic dendritic cells, ↓Th1	↑Chronic infection	^34^^,^^24^
↑*Streptococcus*	↓IFN-β	↓TLR3	↑Acute infection	^4^^,^^40^
↑*Akkermansia muciniphila*, ↑*Bacteroides fragilis*	↑CXCL-11	↑NK, ↑Dendritic cells, ↑IFN-α, ↑IFN-γ	↑ALT	^55^^,^^50^
↓Firmicutes	↑CXCL12-CXCR4	↑Proliferation and migration of immune cells	↑Hepatocellular carcinoma, ↓Cirrhosis	^35^^,^^54^
**HCV infection**				
**Altered microbiome**	**Biomarkers**	**Immunological outcome**	**Clinical aspects**	**References**
↓*Bilophila spp*., ↓*Clostridium IV spp*.	↓IFN-γ	↓TCD8^+^	↑Persistent infection	^2^^,^^41^
↑*Coriobacteriaceae*, ↓*Streptococcus*	↑IL-1β, ↑IL-18	↑Neutrophils, ↑NK	↑Hepatocellular carcinoma	^3^^,^^45^
↑*Prevotella*, ↑*Porphyromonas*	↑IL-6, ↑IL-10, ↑IL-11	↑JAK/STAT3, ↓Apoptosis	↑Hepatocellular carcinoma	^15^^,^^45^
↑*Enterobacteriaceae*, ↓*Pediococcus*	↑IFN-α	↓IL-12, ↓TNF-α	↑Persistent infection	^13^^,^^52^
↑*Klebsiella*	↑IL-22	↑STAT3	↑Tumor growth	^17^^,^^54^
↓*Lactobacillus*	↑TNF-α	↑Hepatic macrophages, ↑Pro-IL-1β	↑Acute hepatic inflammation	^33^^,^^47^
↓*Akkermansia*, ↓*Faecalibacterium*	↑IFN-γ	↑T-cell lymphotoxins	↑Hepatocellular carcinoma	^34^^,^^45^
↑*Burkholderia*, ↑*Megasphaera*	↑TGF-β	↑HLA-E, ↓NK	↑Persistent initial infection	^35^^,^^52^
↑Proteobacteria, ↓*Lachnospiraceae*	↑IL-7	↑Memory T Cells	↑Chronic infection	^36^^,^^51^
↑*Fusobacterium nucleatum*, ↑*Enterococcus faecalis*	↓TGF-β	↓IL-23R Crackdown	↑Dysfunction in the liver	^68^^,^^53^
↑*Helicobacter Pylori*, ↑*Campylobacter jejuni*	↑CXCL12-CXCR4	↑Proliferation and migration of immune cells	↑Hepatocellular carcinoma, ↓Cirrhosis	^78^^,^^54^
↑*Enterobacter*, ↑*Helicobacter*	↑IL-1α	↑NF-kB, ↑Phenotype M2	↑Tumor Promotion	^77^^,^^44^
↑*E. coli*, ↑*Enterococcus faecium*	↑IL-17	↑Neutrophil infiltration	↑Hepatic inflammation	^47^^,^^44^
↓Clostridiales	↑CXCL-10	↑Monocytes, ↑T lymphocytes, ↑ NK	↑Progression of inflammation	^57^^,^^45^
↑*Pseudomonas aeruginosa*	↑CXCL-1	↑B Cells, ↑T lymphocytes	↑Inflammatory condition	^31^^,^^45^
↑Firmicutes, ↑Bacilli	↑CCL7	↑Neutrophils, ↑NK, ↑Monocytes	↑Persistent infection	^18^^,^^45^
↑*Veillonelaceae*	↑IL-11	↑JAK/STAT3, ↓Apoptosis	↑Tumor aggressiveness	^36^^,^^45^
↑*Lactobacillus plantarum*, ↑*Gemella*	↑IL-12	↑Th1 Differentiation	↑Acute infection	^13^^,^^51^
↑*Burkholderia*, ↓*Megamonas*	↑IL-15	↑NK Cytotoxicity	↑Acute infection	^35^^,^^51^
↓*Pediococcus*, ↓*Weissella*	↑IL-7	↑Memory T Cell Renewal	↑Chronic condition	^33^^,^^51^
↓Actinobacteria	↑IL-28, ↑IL29	↑BDCA3, ↑Myeloid cells, ↓IL-12	↑Late chronic condition	^57^^,^^52^
↑*Micrococcus spp*., ↓*Shigella spp*.	↑CCL2, ↑CCL5	↑TCD8^+^, ↑NK	↑Early hepatocellular carcinoma	^2^^,^^54^

Plasmacytoid dendritic cells are also specialized in recognizing viruses and bacteria, they orchestrate an important role in inducing cytotoxicity by NK via IFN-α production suppressing the proliferation of enteropathogens. Barrier function in the gut microbiota is associated with the translocation of pro-inflammatory products such as lipopolysaccharides, short-chain fatty acids, related to increased levels of circulating inflammatory markers such as IL-1, IL-1RN, IL-8, IL-13, IL-18, C-reactive protein, TNF, and TGF-β.^35^

Cathepsin protease of enteropathogens such as bacteria and viruses alters the microbiota, and may promote the release of pathogens, followed by the translocation of the human epithelium. As a result, the production of pro-inflammatory mediators such as CXCL-8 is increased, the bacteria-derived ATP may limit the secretory response of IgA in the small intestine, affecting the homeostasis of intestinal commensal bacteria, including amount and composition of polysaccharide A. *Bacteroides fragilis* is able to upregulate TLR2 expression on the surface of Dendritic Cells (DCs), promoting IL-10 secretion from TCD4^+^ lymphocytes and differentiation of TCD4^+^ naive cells into Th1 and Th2.^36^

Hepatic macrophages lead to the release of TNF-α, the recognition of bacterial products by TLRs and NLRs, and the induction of cytokine transcription by the altered microbiota in the gut-liver axis (Table 1), however, constant exposure makes cells refractory to TLR stimulation, also leading to active immune suppression via IL-10 or TGF-β cytokines. The inflammasome complex requires a signal to induce the inflammatory response and leads to the activation of caspase-1, which proteolytically activates cytokines. This inflammatory response is amplified by IL-1b, which in turn provides positive advancement for pro-inflammatory molecules.^37^

Stimulation of naive T-lymphocytes by antigen-presenting cells matured by PAMPs, such as CDs, leads to the generation of distinct immune cell responses that determine the outcome of antigen exposure through induction of cytotoxic lymphocytes TCD8^+^, TCD4^+^, Tregs, Th (Th1, Th2, and Th17), as well as unconventional Tγδ lymphocytes. Among these cell subsets, Th17 lymphocytes and Tγδ can directly influence the functions of the intestinal epithelial barrier. Consequently, the balance between the proportions of cytokines of the epithelial protective barrier (IL-22, IL-17 and IL-33), pro-inflammatory cytokines (IL-1β, TNF-α, IL-2, IL-6, IL-15, IL-21 and IL-23) and anti-inflammatory (TGF-β and IL-10) can determine the inflammatory or homeostatic state of the intestine.^38^

Infection with intracellular pathogens drives the development of Th1 cells, TCD4^+^ lymphocytes can also adopt an anti-inflammatory phenotype, with the specific transcription of Treg cells that control unwanted activation of the immune system. In addition to thymus-derived TCD4^+^ Foxp3 lymphocytes, several subsets of cells can be generated in the gut from naive T-cells, some of which produce IL-10, and several commensals such as Bifidobacteria infantis and *Faecalibacterium prausnitzii* have been shown to induce the production of Foxp3+Tregs and IL-10 in the gut.^39^

Intestinal epithelial cells use microbial cell recognition to adjust metabolic homeostasis. IL-18 also plays an important role in this process, its secretion requires activation through TLRs or G protein-coupled receptor and post-transcriptional cleavage through the NLRP3 inflammasome, which can be induced by IFNs. IL-18 and IL-22 derived from immune cells help regulate the antimicrobial effect, and the CCL20 protein generates a cellular response that includes cytokine secretion, autophagy, epithelial regeneration, and production of antimicrobial agents.^40^

### Dysregulated cytokines in the microbiome of HBV and HCV infection

HBV impairs IFN production by inhibiting its synthesis in human hepatocytes, the RLH (Helicases) mediated signaling pathway plays a critical role in IFN-β induction, but the mechanism by which HBV disrupts RLH-mediated IFN-β cytoplasmic induction in human hepatocytes is still poorly understood. The IRF3 factor is able to boost IFN-β production, but to avoid immune recognition and response, viruses orchestrate mechanisms to directly target molecules critical in IFN-β induction, resulting in an uncontrolled microenvironment, as in the case of HCV's NS3–4A serine protease, which can cleave TRIF and IPS-1, leading to acquired IFN-β deficiency in infected cells and altering the microbiome.^41^

TCD8^+^ cells and their function in humans with HBV carriers is seriously impaired, with decreased activation receptor, increased inhibitory receptor and decreased production of IFN-γ and TNF-α cytokines, a state known as exhaustion, HBcAg suppresses IFN-β expression, TCD8^+^ cell exhaustion is sustained by IL-10 secretion by interaction with HBcAg. In the case of the central HCV protein, it acts as a ligand for TLR2 in human kupffer cells, inducing the production of cytokines such as IL-10, and the blockade of TLR2 by the pathophysiology of HBV or HCV, being able inhibit the production of IL-10, overexpressed levels of this cytokine are also common in the pathogenesis of infection.^42^

IL-35 dysregulation may have multifunctional roles in the pathogenesis of infection, its stimulation may reduce the concentrations of IFN-γ, IL-1β, IL-6 and IL-8 produced by peripheral blood mononuclear cells, indicating antiviral immunity inhibited by IL-35 in HBV infection, with a reduction in the secretion of pro-inflammatory cytokines (Table 1) in peripheral blood mononuclear cells co-stimulated by HBsAg and IL-35, decreased STAT1 phosphorylation also occurs.^43^ In the case of HCV infection, elevated levels of IL-1β and IL-18 correlate with chronic infection, the virus can inhibit the maturation of dendritic cells and increase the production of IL-10, where higher levels have been associated with HCV persistence in chronic patients, IL-1β and IL-18 mediated by HCV also have the ability to activate quiescent stellate cells towards fibrosis.^44^

Unresolved inflammation results from the persistence of initial stimuli or deficiency in immune mechanisms, its main features include infiltration of immune cells such as HC-associated macrophages, immature myeloid cells and T-cells. The imbalance of pro-inflammatory cytokines such as TNF-α, IL-6, IL-1 and anti-inflammatory cytokines such as IL-10, IL-12 and TGF-β, with the occurrence of angiogenesis and tissue remodeling cause inflammation during the development of HC. Oncogenic RAS has been shown to transcriptionally regulate pro-inflammatory cytokines IL-8, IL-6, CXCL-1, and CXCL-2 contributing to the tumorigenesis support medium in the microbiome.^45^

The persistence of this cycle in the pathobiome in turn leads to more genetic mutations and dysregulation of IL-1β, IL-6, IL-10, IL-12, IL-13, IL-18, and TGF-β. Inflammasome can lead to liver damage, steatosis, inflammation, and fibrosis through the activation of pro-inflammatory cytokines IL-1α, IL-1β, and TNF-α. On the other hand, the increased production of cytokines IL-1β, IFN-γ and IL-6, by triggering the expression of lymphotoxin heterodimers from T-cells and dendritic cells promotes HC. The activation of NF-kB triggers the release of CXCL-10, CXCL-1 and CCL7, which attracts monocytes, T/B lymphocytes, neutrophils and NK, the progression of this inflammatory process favors hepatitis and HC.^46^

The microbiota is involved in chronic inflammation with expression of IL-8, IL-29 and Cyclooxygenase 2 (COX-2) in HBV infection, IL-8 is stimulated by HBV proteins, and IL-8 expression is higher in chronic patients when compared to the control group, with the exacerbation of liver injury this cytokine increases its expression contributing to HC. IL-29 belongs to the IFN-λ gene family, the axis of these anti-inflammatory cytokines IL-29/IL-8/COX-2, provides upregulation and negative feedback, when it is dysregulated the expression of IL-8 alters the antiviral activity of IL-29 favoring persistent HBV infection, but also induces high expression of pro-inflammatory factor COX-2.^47^

Inflammasomes are mainly formed in myeloid cells and their main function is the cleavage of pro-IL-1β and pro-IL-18 into their active forms IL-1β and IL-18. In the case of HCV infection in the microbiome, TNF-α serves as the starting factor in hepatic macrophages, leading to NF-kB activation and production of pro-IL-1β, the central protein of HCV, directs intracellular calcium mobilization to transmit NLRP3 inflammasome assembly through phospholipase C signaling and phosphorylation, leading to more IL-1β production from macrophages, thus establishing increased inflammation in the liver microbiome.^48^

**Profile of inflammatory cytokines in hepatocarcinogenesis of HBV and HCV**

TNF-α and IFN-γ synergistically induce the expression of IL-32, which in turn suppresses HBV by downregulating transcription factors enriched through ERK1 activation, among members of the Tripartite Motif Family (TRIM) have been shown to be one of the most strongly induced by IFN-α and IFN-γ in HepG2 cells and several members of the TRIM family are antiviral performers. It is known that HBeAg can induce the secretion of IL-10 by Tregs and this cytokine is responsible for NK dysfunction, characterized by the expression of inhibitory receptor NK group 2, in addition to HBV affecting the production of IL-12 by dendritic cells and attenuating the production of IFN-γ by NK.^49^

IL-22 has been shown to play a role in promoting tissue repair in the inflammatory environment, however IL-22 has also been shown to play a pathological effect in exacerbating chronic inflammation and liver injury, its expression has been implicated in the regulation of different sets of liver diseases. It is known that IL-22 has a pro-inflammatory effect in the presence of IL-17 and can be regulated by IL-17, the increase of Th17 cells in HBV-infected hepatocytes, produces more IL-22, forming a positive feedback loop, which promotes fibrosis and liver disease. In combination, T-cell IFN-γ actively recruits liver macrophages to produce fibrosis-promoting cytokines in the early phase of chronic liver disease.^50^

High levels of IL-10 and TGF-β may impair the ability of T-lymphocytes to expand, attenuating viral control, in patients with HBV CXCL-9 induction is restricted to IFN-γ and this cytokine works predominantly to recruit TCD8^+^ lymphocytes, in contrast CXCL-10 is strongly induced by IFN-γ and IFN-α/β, being responsible for NK recruitment, Th1 and TCD8^+^ effectors. Hepatocytes produce CXCL-9 or CXCL-10, to attract NK, dendritic and T memory cells, an increase in the levels of these cytokines is reported in HBV crises, both acute and chronic patients have an increase in CXCL-9, CXCL-10 and CXCL-11 at peak alanine transaminase levels.^51^

In HCV infection, IFNs act on hepatocytes and dendritic cells through TLR3, TLR7, TLR9 and induce IL-12 production, which supports Th1 differentiation and subsequent IFN-γ production in the hepatocarcinogenic microenvironment. IL-18 and IL-27 from dendritic cells can synergize with IL-12 to promote Th1 development, IL-12 and IL-15 from dendritic cells also contribute to the cytotoxicity and survival of NKs. HCV-specific TCD4^+^ cells have lower expression of alpha-chain IL-7 receptor, given the importance of IL-7 in the survival and renewal of memory T-cells, the loss of this signal contributes to depletion in the response of TCD4^+^ cells in HCV infection.^52^

Stimulation of TLR2 during HCV infection leads to production of TNF-α, IL-6, IL-8 and TLR2 expression by peripheral blood mononuclear cells is increased in HCV infection, with plasmacytoid dendritic cells being stimulated by infected cells via TLR7 and secreting large amounts of IFN-α/β. Monocytes and macrophages are the main mediators of the inflammatory response during HCV infection, where the overproduction of TNF-α, IL-1, IL-10 and TGF-β influences the course of infection. TLR-mediated IL-12 and IL-18 from liver-resident macrophages activate NK to produce IFN-γ, inducing TLR4 expression, increasing IL-4 and IL-6 secretion from B-lymphocytes and hepatocytes in hepatocarcinogenesis.^53^

The levels of IL-1β, IL-8 and TGF-β tend to be more severe hepatitis C, along with correlated positive results of TGF-β, TNF-α, IL-1β and IL-8. The frequency of intrahepatic Th17 lymphocytes was correlated with the expression of IL-8 where an association with the severity of fibrosis was observed, it was noted that at low concentrations TGF-β synergizes with IL-6 and IL-21 promoting Th17 differentiation in the HC microenvironment. The absence of hepatic expression of IL-10 and TGF-β, in addition to the frequency of activation of Tregs, may be due to several activation states in cases with different severities, other factors contribute to the limitation of inflammation such as IL-21, already observed attenuating Th17, Treg, TCD8^+^ lymphocytes and B lymphocytes.^54^

Carcinogenesis is associated with persistent production of cytokines that stimulate many liver cell types, a predominant role of Th2-like cytokines (IL-4, IL-8, IL-10 and IL-5) compared to Th1-like cytokines (IL-1α, IL-1β, IL-2 and TNF-α) in the microenvironment has been associated with a more aggressive phenotype in HC. Higher levels of IL-22 were detected in HC, leading to tumor growth, inhibition of apoptosis, and promotion of metastasis due to activation of STAT3. IL-10 upregulation is also present in HC tumors and higher levels of IL-2 and IL-15 in peritumoral tissue, with the CXCL-12-CXCR-4 chemokine axis important in angiogenesis being overexpressed in HC compared to cirrhosis.^55^

**Microbiota as a potential inducer of liver inflammation**

The gut microbiota is a key determinant of inflammation, it also plays a key role in chronic inflammatory disease in the liver, the essential role seems to largely reflect that gut microbial products that activate TLR/NLR drive disease-defining inflammation. Although the development of the disease is dictated by the genetics of the host, as well as a variety of environmental, behavioral, and infection factors, the mechanisms by which all of these factors affect susceptibility to the disease can be viewed from the prism of inflammation. In most, if not all liver diseases, are associated with elevated markers of inflammation, especially cytokines, which are thought to play a role in driving disease.^16^

The role of the microbiota (Table 1) in hepatocarcinogenesis is primarily driven by inflammatory pathways, which are initiated by variations between gut bacteria, the immune system and the liver. The process involves the interaction of macrophages, Kupffer cells and PAMPs in the elimination cascade of microorganisms; most macrophage populations with Kupffer cells respond with low concentrations of PAMPs, endotoxin or lipopolysaccharides, through the activation of NF-kB by binding to TLRs and NOD receptors. This mechanism consequently leads to an inflammatory chain reaction and promotes inflammation and cytokine release, causing hepatocyte apoptosis.^56^

Changes in the gut microbial community profile are associated with cirrhosis and its complications such as hepatic encephalopathy, spontaneous bacterial peritonitis, and other infections. The microbial community can act in the regulation of the metabolic balance of a whole body, the ratio between Firmicutes and Bacteroidetes at normal levels indicates the health status of the host, when these microbes are altered in the microbiota after HBV infection, there can be the translocation of bacteria to the intestine, with a higher proportion in the members of *Porphyromona, Treponema, Eubacterium, Solobacterium, Filifactor, Fusobacterium, Parvimonas* and *Pseudomonas*.^57^

Among the intestinal representatives, bacteria are classified into beneficial, which are effective in maintaining health and preventing aging, such as digestion/absorption and boosting immunity, typical bacteria that include Bifidobacteria, lactic acid bacteria, and harmful bacteria with adverse effects on the body that include Bacilli, *Staphylococcus*, toxic *E. coli*, and dysbiosis is associated with their pathogenesis in many diseases. Reports on the microbiome of HCV patients have mainly highlighted a decrease in the diversity of constituent species, while others have reported an increase, such as in HBV infection several differences such as ethnicity, stage of the disease are suggested to affect changes in the gut-liver axis.^4^

In patients with hepatitis a profound alteration in the gut microbiota is reflected by the significantly enriched *Actinomyces, Clostridium sensu stricto, Megamonas, Lachnospiraceae* and a concomitant decrease in *Alistipes, Bacteroides, Asaccharobacter, Parabacteroides, Butyricimonas, Clostridium IV, Coriobacteriaceae, Escherichia, Shigella, Ruminococcus*, Clostridiales, *Enterobacteriaceae, Lachnospiraceae* and *Ruminococcaceae*. The course of liver disease in HBV/HCV infection may also be reflected by commensal microbiota profiles.^58^

**Role of host microbiota in susceptibility to viral infections and pathogenesis**

In addition to the resident microbiota, several bacteria pass transiently as a consequence of ingestion, in the intestinal tract a single layer of epithelial cells forms a physical barrier between the intestinal lumen, the lamina propria and the mucosa-associated lymphoid tissue, the mucus secreted by goblet cells in the epithelium serves to compartmentalize especially the bacteria of the lumen and prevent bacterial colonization of the epithelium. When inflammation occurs, this barrier is impaired, cytokines make the epithelium permeable, which can favor the entry and replication of viral agents.^59^

Many of the liver diseases are linked to an imbalance in microbial communities, over host immunity, which implies that dysbiosis is characterized by an underlying impairment of immune functions, which regulate microbiota and microbial metabolism. Measurements within the normal range may indicate homeostasis, while values outside this range may indicate dysbiosis. The metabolites produced by the colon microbiota profoundly influence host physiology, which makes changes in the microbial metabolite profile a common contributor to disease, infection, and co-infection.^60^

Microbial density and composition are affected by chemical, nutritional, and immune gradients throughout the gut. There are usually high levels of acids, oxygen, antimicrobials and these properties limit microbial growth so that only fast-growing facultative anaerobes are able to adhere to the epithelium survive. There is a different microbial composition among the various gastrointestinal organs, which is an apparent characteristic in periods of localized inflammation. It is proposed that interindividual differences in species composition may favor the clinical profile of the individual.^61^

Accumulating evidence suggests that, in addition to PAMPs, nutrition and bacterial metabolites may have a major impact on the immune response in the gut. Short-chain fatty acids from the gut microbiota participate in the regulation of various body systems through their metabolites, mainly fatty acids absorbed into the bloodstream to reach other organs. Thus, the modulation of the intestine, nervous, endocrine or blood system can be influenced by these microbial molecules, where imbalances in the microbiota tend to cause metabolic disorders and susceptibility to infectious agents.^62^

Some pathogens can evade the immune system through various mechanisms, causing persistent infections, which can lead to latent lifelong infections, unlike an acute infection, in a persistent infection the pathogen is not eliminated, the pathogen's genome, or the pathogen-derived proteins continue to be produced for long periods. An altered microbiota, skin barriers, ruptured mucous membranes, allow microorganisms to have the opportunity to establish infections or co-infections such as EBV, HIV, HBV and HCV.^63^

### HBV and HCV co-infection in the pathobiome

HBV and HCV share modes of transmission and their combined infection is a fairly frequent occurrence, particularly in areas where the viruses are endemic, this co-infection is considered a key factor that favors the progression of liver fibrosis and the establishment of cirrhosis.^64^ In particular, when dysbiosis occurs in the gastrointestinal tract, it can lead to the development of benign and malignant tumors, in relation to HBV and HCV co-infection, in the pathobiome, the bacteria end up negatively influencing to cause tumorigenesis. Among other predominant microbes that cause carcinogenesis are Bacteroidetes, Firmicutes, Proteobacteria, with species of *Bacteroides fragilis, Clostridium septicum, E. coli, Enterococcus faecalis, Fusobacterium spp, H. pylori* and *Streptococcus bovi*.^65^

Dysbiosis through the gut-liver axis leads to progression to severe forms of liver failure in HBV and HCV co-infection, in this co-infection added to the dysbiosis picture an increase in *Enterobacteriaceae* and a loss of Bifidobacterium is noted. It was possible to analyze that α diversity, which is the richness of species with a positive function in the microbiome, decreases significantly in patients with HC, and HBV and HCV co-infection is related to the progression of cirrhosis associated with increased intestinal permeability, bacterial overgrowth of the small intestine and bacterial translocation. This situation allows microbe-derived compounds to access the liver through the hepatic portal circulation, stimulating the production of pro-inflammatory cytokines, which promote liver inflammation, fibrinogenesis, and cirrhosis.^35^

## Discussion

The physiopathogenesis of HBV modulated the response of T-lymphocytes in HC, it is noted that TGF-β signaling is associated with tumor immune infiltration, or depleted adaptive immune responses. Regarding HC, the heterogeneity of tumor-infiltrating leukocytes was observed, indicating an immunosuppressive tumor microenvironment, enriched with exhausted TCD8^+^ cells, Tregs, targeting different HBV-derived epitopes.^66^ In the case of HCV pathophysiogenesis, the most significant molecular pathways altered in HCV infection include TERT, β-catenin, p53, PI3K-mTOR and ECH/NRF2-kelck. The central HCV protein can trigger angiogenesis through a mechanism that involves the cross-response between the expression of the cytokine TGF-β2, with the VEGF and CD34 pathways.^67^

The genotypes of HBV/HCV, have a significant influence on the expression of cytokines, the HBV genotype C, is related to mutations that reach regions restricted to the major histocompatibility complex II, showing that these variants have a direct influence on HC, conferring immune escape against TCD4^+^ lymphocytes.^10^ Genotype 1b HCV is more prevalent in HC, being associated with earlier hepatitis C, with a worse response to treatment.^11^ The genetic heterogeneity of these viruses guides changes in the proportion of cytokines, altering the microbiome, generating dysregulation in the proportion of IL-2, IL-10, TGF-β and contributing to alterations in the intestine-liver axis.^9^

In HBV/HCV infection, the genotypes of these viruses have the ability to cause T-cell exhaustion; this mechanism prevents the proper signaling of TCD4 lymphocytes, favoring the development of secondary infections by opportunistic enteropathogens. Some microbial representatives involved belong to the taxon *Neisseria, Sphingomonas, Enterobacteriaceae* and *Campylobacter*, related to dysbiosis, by infection of HBV/HCV.^5^ This genetic heterogeneity of HBV/HCV, directs super pathogenicity, with a direct effect on the intestine-liver axis, via immune system dysfunction.^12^

Cytokines are communication molecules between the immune system, and bacteria have developed several methods to modulate the ability of cells to produce these molecules, such as *Lacticaseibacillus* with lactocepine which is a protease capable of degrading IP-10 (CXCL-10), or *Eggerthella lenta* which produces a Cgr2 enzyme that also degrades RORγt, increasing IL-17a levels.^68^ Cirrhosis caused by viral infection has bacterial profiles that include increased numbers of *Prevotella, Streptococcus, Staphylococcaceae, Enterococcus*, and decreased numbers of *Ruminococcus* and *Clostridium*.^35^ With the increase of inflammation in this microbiome, the NLRP6 inflammasome promotes the maturation of IL-1β and IL-18, inducing cell death by pyroptosis, also regulating the expression of antimicrobial peptides, when these cytokines are dysregulated, there is a lack of control in the microbiome.^69^

Altered expressions of IFN-α and IL-8 are observed as pathogenetic markers correlated with viral load in HBV, IFN-α increases the expression of the apoptosis-inducing ligand related to tumor necrosis factor TRAIL in peripheral NK, which could induce hepatocyte apoptosis, while IL-8 has been shown to increase the expression of the TRAIL receptor. CXCL-9 levels are correlated with ALT activity, with CXCL-10 positively correlating with increased ALT and T-cell expression.^70^ In HCV infection it is observed that TCD4^+^ cells have limited functionality as a consequence of IL-2 suppression and a distortion of IL-4, IL-10 response due to IL-12 inhibition mediated by the HCV nucleus.^71^

It is well established that the HBV genes related to the p53 signaling pathway and the cytokines CCL2, CXCL-8, CXCL-9, CXCL-10 were found to be relatively higher in HBV infection, and higher levels of IL-6 were also characteristic in patients with HC.^72^ In HCV infection, the TGF-β signaling pathway is altered, resulting in the progression of liver injury, the tumor suppressor activity of TGF-β changes to fibrogenic leading to the risk of HC, IL-1, IL-23, IL-6 and lymphotoxins were involved in the development and progression of inflammation in HC.^73^

For the few microbes that are regulated in patients with HBV infection, their dysregulation is strongly associated with decreased cellular activity, on the other hand, when upregulation occurs, the increase of cytokines CCL28 and CCL26 are observed, and IL-6 and IL-10 are suppressed in the presence of microbes in the pathobiome of gastrointestinal infection.^74^ In the microbiome of HCV patients, it is noted that there is limited antiviral impact on NK activity, with its inability to secrete IFN-γ, where macrophages engulfing HCV lead to the release of cytokines IL-6 and IL-1β in a surrounding microenvironment and apoptosis of cells infected with the NS5A protein increases the release of IL-10, and the suppression of IL-12 in the chronic microbiome.^75^

In the disease of the gut microbiome associated with viral infections, dysbiosis ends up causing failures, as the *Clostridium difficile* that exists in the normal commensal microbiota ends up becoming pathogenic, which ends up damaging the cytoskeleton and the normal epithelial barrier, inducing aberrant inflammatory response and cell death. Another characteristic in HBV and HCV infections is the reduction of *Faecalibacterium prausnitizii* and *Roseburia spp*. These are butyrate-producing bacteria, which favors local inflammation by decreasing the action of cytokines.^76^ In dysbiosis caused by HBV and HCV infection, a number of increasing metabolites can influence the immune system, such as tryptophan metabolites in *Lactobacillus reuteri* that serve as ligands for host receptors, including the aryl hydrocarbon receptor, which promotes the transcription of IL-22, when dysregulated provides a deficiency in the protection of the mucosal barrier, promoting inflammation and transition to the hepatic portal axis.^77^

Fibrosis and defective liver function can promote changes in bacterial populations, contributing to HC, as do liver lesions associated with HBV and HCV infection. Recent studies highlight the ability of *Helicobacter hepaticus* to promote aflatoxin-induced HC, so microbial metabolisms play critical roles in the development of liver cancer.^78^ Once intestinal microbial metabolites break through the intestinal barrier and enter the hepatic portal system, the gut-liver axis allows them to damage the liver with consequent inflammation, cytokine production, fibrinogen, and cirrhosis.^37^

The inflammatory mechanism is not specific to bacteria, in almost all mechanisms of bacterial inflammation and cancer, the implicated bacteria reside in the host for many years, accompanying this response to inflammation is the antiviral action of IL-1β, TNF-α and IFN-γ. This response may be accompanied by IL-8, IL-2, CXCL-9 and CXCL-10 to the site of inflammation, with excess immune overstimulation by persistent antigens creating B-lymphocyte hyperplasia, triggered by T-cells, which can lead to mucosa-associated lymphoid tissue lymphomas.^79^

In the microenvironment of hepatocarcinogenesis of infection, liver damage can occur producing IL-8/CXCL-8, which increases the expression of apoptosis, with IL-10 produced by Th2 cells, inhibiting the function of monocytes, macrophages, T-cells, preventing the production of IL-1, IL-6, IL-8, IL-12 and TNF-α in HBV and HCV co-infection.^79^ Among the bacterial representatives that seriously contribute to this immunological depletion in co-infection are *Bacteroides, Clostridium, Bifidobacterium, Peptostreptococcus, Ruminococcus, Escherichia, Lactobacillus, Enterobacter* and *Enterococcus* in the intestine, that, when altered, contribute to the pathogenesis in the intestine-liver axis, which can lead to hepatocarcinoma.^80^

## Conclusion

Patients infected with HBV and HCV have an unbalanced clinical profile due to dysbiosis acquired in monoinfection or co-infection by these viruses. Bacterial metabolites and toxins are altered as a result of viral hepatitis infection, when this pathological picture is aggravated as a result of microbial products entering the hepatic portal circulation, susceptibility to the development of viral hepatitis or liver diseases is amplified. HBV and HCV can deregulate the microbiota from the normal to the pathological stage, dysbiosis contributes to chronic viral infection and can cause pathogenesis for intermittent periods.

Among the most altered and overexpressed representatives in HBV infection were *Bifidobacterium*, Bacteroides, Proteobacteria, Actinobacteria, *Faecalibacterium prausnitzii*, Alloprevotella, being these commensals that are dysregulated by the depletion of the immune system related to cirrhosis or chronic hepatitis B. In HCV infection, it was observed that *Enterobacteriaceae*, Firmicutes, Bacilli, *Enterococcus faecium, Helicobacter pylori, Burkholderia*, were overexpressed. Patients co-infected with HBV and HCV have more severe cases of the disease, with higher risks of liver damage, cirrhosis or HC.

It was possible to observe that dysregulated cytokines amplified the development of hepatocarcinogenesis, infiltrates of T-lymphocytes can secrete lymphotoxins, with the alteration of the microbiota, this mechanism becomes persistent, in addition to HBV and HCV deregulating the function of Tregs altering the immune homeostatic control. It is noted that TLR4 is targeted by bacterial pathogens and the translocation of these pathogens in the gut contributes to the overexpression of HBV and HCV in the hepatic tumor microenvironment. Altered cytokines induce excessive or persistent responses, which may favor microlesions and consequent hepatocarcinogenesis by microbial metabolites via the hepatic portal system.

## Conflicts of interest

The authors declare no conflicts of interest.

## References

[1] Spanu D, Pretta A, Lai E, Persano M, Donisi C, Mariani S (2022). Hepatocellular carcinoma and microbiota: implications for clinical management and treatment. World J Hepatol.

[2] Neag MA, Mitre AO, Catinean A, Buzoianu AD (2021). Overview of the microbiota in the gut-liver axis in viral B and C hepatitis. World J Hepatol.

[3] Milosevic I, Russo E, Vujovic A, Barac A, Stevanovic O, Gitto S (2021). Microbiota and viral hepatitis: state of the art of a complex matter. World J Hepatol.

[4] Mizutani T, Ishizaka A, Koga M, Tsutsumi T, Yotsuyanagi H. (2022). Role of microbiota in viral infections and pathological progression. Viruses.

[5] Lv Z, Xiong D, Shi J, Long M, Chen Z. (2021). The interaction between viruses and intestinal microbiota: a review. Curr Microbiol.

[6] Katze MG, He Y, Jr MG. (2022). Viruses and interferon: a fight for supremacy. Nature Reviews Immunology.

[7] Castaneda D, Gonzalez AJ, Alomari M, Tandon K, Zervos XB. (2021). From hepatitis A to E: a critical review of viral hepatitis. World J Gastroenterol.

[8] Tian Z, Xu C, Yang P, Lin Z, Wu W, Zhang W (2022). Molecular pathogenesis: connections between viral hepatitis-induced and non-alcoholic steatohepatitis-induced hepatocellular carcinoma. Front Immunol.

[9] Rehermann B. (2013). Pathogenesis of chronic viral hepatitis: differential roles of T cells and NK cells. Nat Med.

[10] Sunbul M. (2014). Hepatitis B virus genotypes: global distribution and clinical importance. World J Gastroenterol.

[11] Zein NN. (2000). Clinical significance of hepatitis C virus genotypes. Clin Microbiol Rev.

[12] Campbell DE, Li Y, Ingle H, Baldridge MT. (2023). Impact of the microbiota on viral infections. Annu Rev of Virol.

[13] Xu D, Huang Y, Wang J. (2015). Gut microbiota modulate the immune effect against hepatitis B virus infection. Eur J Clin Microbiol Infect Dis.

[14] El-Mowafy M, Elgaml A, El-Mesery M, Sultan S, Ahmed TAE, Gomaa AI (2021). Changes of gut-microbiota-liver axis in hepatitis C virus infection. Biology (Basel).

[15] Lu H, Wu Z, Xu W, Yang J, Chen Y, Li L (2011). Intestinal microbiota was assessed in cirrhotic patients with hepatitis B virus infection. Microb Ecol.

[16] Chassaing B, Etienne-Mesmin L, Gewirtz AT. (2013). Microbiota-liver axis in hepatic disease. Hepatology..

[17] Chu H, Khosravi A, Kusumawardhani IP, Kwon AHK, Vasconcelos AC, Cunha LD (2016). Gene-microbiota interactions contribute to the pathogenesis of inflammatory bowel disease. Science.

[18] Schnabl B, Brenner DA. (2014). Interactions between the intestinal microbiome and liver diseases. Gastroenterology.

[19] Dapito DH, Mencin A, Gwak G-Y, Pradere J-P, Jang M-K, Mederacke I (2012). Promotion of hepatocellular carcinoma by the intestinal microbiota and TLR4. Cancer Cell.

[20] Sd Ryder (2001). Beckingham. Acute hepatitis. BMJ.

[21] Torre P, Aglitti A, Masarone M, Persico M. (2021). Viral hepatitis: milestones, unresolved issues, and future goals. World J Gastroenterol.

[22] Michelsen PP, Francque SM, van Dongen JL. (2005). Viral hepatitis and hepatocellular carcinoma. World J Surg Oncol.

[23] Trehanpati N, Ak Vyas (2017). Immune regulation by T regulatory cells in hepatitis B virus-related inflammation and cancer. Scand J Immunol.

[24] Rizzo GEM, Cabibbo G, Craxi A. (2022). Hepatitis B virus-associated hepatocellular carcinoma. Viruses.

[25] Fisicaro P, Barili V, Rossi M, Montali I, Vecchi A, Acerbi G (2020). Pathogenetic mechanisms of T cell dysfunction in chronic HBV infection and related therapeutic approaches. Front Immunol.

[26] Blackard JT, Shata MT, Condado NJ, Sherman KE. (2008). Acute hepatitis C virus infection: a chronic problem. Hepatologia.

[27] Ferri C, Sebastiani M, Giuggioli D, Colaci M, Fallahi P, Piluso A (2015). Hepatitis C virus syndrome: a constellation of organ- and non-organ specific autoimmune disorders, B-cell non Hodgkin's lymphoma, and cancer. World J Hepatol.

[28] Nakagawa H, Maeda S. (2012). Inflammation- and stress-related signaling pathways in hepatocarcinogenesis. World J Gastroenterol.

[29] Hoshida Y, Fuchs BC, Bardeesy N, Baumert TF, Chung RT. (2014). Pathogenesis and prevention of hepatitis C virus-induced hepatocellular carcinoma. J Hepato.

[30] Mitchell J, Lemon SM, McGivern DR. (2015). How do persistent infections with hepatitis C virus cause liver cancer?. Curr Opin Virol.

[31] Wróblewska A, Bielawski KP, Sikorska K. (2021). Occult infection with hepatitis c virus: looking for clear-cut boundaries and methodological consensus. J Clin Med.

[32] Valentini M, Piermattei A, Di Sante G, Migliara G, Delogu G, Ria F (2014). Immunomodulation by gut microbiota: role of toll-like receptor expressed by T cells. J Immunol Res.

[33] Ashfaq UA, Iqbal MS, Khaliq S. (2016). Role of toll-like receptors in hepatitis C virus pathogenesis and treatment. Crit Rev Eukaryot Gene Expr.

[34] Sehgal R, Bedi O, Trehanpati N. (2020). Role of microbiota in pathogenesis and management of viral hepatitis. Front Cell Infect Microbiol.

[35] Harper A, Vijayakumar V, Ouwehand AC, Ter Haar J, Obis D, Espadaler J (2021). Viral infections, the microbiome, and probiotics. Front Cell Infect Microbiol.

[36] Wang Y, Pan CQ (2019). Xing H. Advances in gut microbiota of viral hepatitis cirrhosis. Biomed Res Int.

[37] Zamparelli MS, Rocco A, Compare D, Nardone G (2017). The gut microbiota: a new potential driving force in liver cirrhosis and hepatocellular carcinoma. United European Gastroenterol J.

[38] Fakhariam F, Thirugnanam S, Welsh DA, Kim W-K, Rappaport J, Bittinger K (2023). The role of gut dysbiosis in the loss of intestinal immune cell functions and viral pathogenesis. Microorganisms.

[39] Lee YK, Mazmanian SK. (2010). Has the microbiota played a critical role in the evolution of the adaptive immune system?. Science.

[40] Thaiss CA, Zmora N, Levy M, Elinav E. (2016). The microbiome and innate immunity. Nature.

[41] Yu S, Chen J, Wu M, Chen H, Kato N, Yuan Z. (2010). Hepatitis B virus polymerase inhibits RIG-I- and Toll-like receptor 3-mediated beta interferon induction in human hepatocytes through interference with interferon regulatory factor 3 activation and dampening of the interaction between TBK1/IKKϵ and DDX3. J Gen Virol.

[42] Li M, Sol R, Xu L, Yin W, Chen Y, Zheng X (2015). Kupffer cells support hepatitis B virus–mediated CD8+ T cell exhaustion via hepatitis B core antigen–TLR2 interactions in mice. J Immunol.

[43] Li X, Liu X, Wang W (2021). IL-35: a novel immunomodulator in hepatitis B virus-related liver diseases. Front Cell Dev Biol.

[44] Shrivastava S, Mukherjee A, Ray R, Ray RB. (2013). Hepatitis C virus induces interleukin-1β (IL-1)/IL-18 in circulatory and resident liver macrophages. J Virol.

[45] Yu L-X, Ling Y, Wang H-Y (2018). Role of nonresolving inflammation in hepatocellular carcinoma development and progression. NPJ Precis Oncol.

[46] Wu M-Y, Yiang G-T, Cheng P-W, Chu P-Y, Li C-J. (2018). Molecular targets in hepatocarcinogenesis and implications for therapy. J Clin Med.

[47] Yu Y, Gong R, Mu Y, Chen Y, Zhu C, Sun Z (2011). Hepatitis B virus induces a novel inflammation network involving three inflammatory factors, IL-29, IL-8, and cyclooxygenase-2. J Immunol.

[48] Antushevich H. (2020). Interplays between inflammasomes and viruses, bacteria (pathogenic and probiotic), yeasts and parasites. Immunol Lett.

[49] Hillaire MLB, Lawrence P, Lagrange B. (2023). IFN-γ: a crucial player in the fight against HBV infection?. Immune Netw.

[50] Zhao J, Zhang Z, Luan Y, Zou Z, Sun Y, Li Y (2014). Pathological functions of interleukin-22 in chronic liver inflammation and fibrosis with HBV infection by promoting Th17 cell recruitment. Hepatology.

[51] Chang M-L, Liaw Y-F. (2022). Hepatitis B Flare in hepatitis B e antigen-negative patients: a complicated cascade of innate and adaptive immune responses. Int J Mol Sci.

[52] Dustin LB. (2017). Innate and adaptive immune responses in chronic HCV infection. Curr Drug Targets.

[53] Saha B, Szabo G. (2014). Innate immune cell networking in hepatitis C virus infection. J Leukoc Biol.

[54] Rios DA, Casciato PC, Caldirola MS, Gaillard MI, Giadans C, Ameigeiras B (2021). Chronic hepatitis C pathogenesis: immune response in the liver microenvironment and peripheral compartment. Front Cell Infect Microbiol.

[55] Hernandez-Gea V, Toffanin S, Friedman SL, LIovet JM. (2013). Role of the microenvironment in the pathogenesis and treatment of hepatocellular carcinoma. Gastroenterology.

[56] Gupta H, Youn GS, Shin MJ, Suk KT. (2019). Role of gut microbiota in hepatocarcinogenesis. Microorganisms.

[57] Ling Z, Liu X, Cheng Y, Jiang X, Jiang H, Wang Y (2015). Decreased diversity of the oral microbiota of patients with hepatitis b virus-induced chronic liver disease: a pilot project. Sci Rep.

[58] Li N, Ma T-W, Pang M, Fan Q-L, Hua J-L. (2019). The commensal microbiota and viral infection: a comprehensive review. Front Immunol..

[59] Bron PA, Kleerebezem M, Brummer R-J, Cani PD, Mercenier A, MacDonald TT (2017). Can probiotics modulate human disease by impacting intestinal barrier function?. Br J Nutr.

[60] Lee J-Y, Tsolis RM, Bäumler AAJ. (2022). The microbiome and gut homeostasis. Science.

[61] Thurby E, Juge N. (2017). Introduction to the human gut microbiota. Biochem J.

[62] Sun M, Wu W, Liu Z, Cong Y. (2017). Microbiota metabolite short chain fatty acids, GCPR, and inflammatory bowel diseases. J Gastroenterol.

[63] Thakur A, Mikkelsen H, Jungersen G. (2019). Intracellular pathogens: host immunity and microbial persistence strategies. J Immunol Res.

[64] Raimondo G, Cacciamo G, Saitta C. (2005). Hepatitis B virus and hepatitis C virus co-infection: additive players in chronic liver disease?. Ann Hepatol.

[65] Akbar N, Khan NA, Muhammad JS, Siddiqui R. (2022). The role of gut microbiome in cancer genesis and cancer prevention. Health Sci Rev.

[66] You M, Gao Y, Fu J, Xie R, Zhu Z, Hong Z (2023). Epigenetic regulation of HBV-specific tumor-infiltrating T cells in HBV-related HCC. Hepatology.

[67] Dash S, Aydin Y, Widmer KE (2020). Nayak L. hepatocellular carcinoma mechanisms associated with chronic HCV infection and the impact of direct-acting antiviral treatment. J Hepatocell Carcinoma.

[68] Hitch TCA, Hall LJ, Walsh SK, Leventhal GE, Slack E, Wouters T (2022). Microbiome-based interventions to modulate gut ecology and the immune system. Mucosal Immunol.

[69] Zheng D, Liwinski T, Elinav E. (2020). Interaction between microbiota and immunity in health and disease. Cell Res.

[70] Kramvis A, Chang K-M, Dandri M, Farci P, Glebe D, Hu J (2022). A roadmap for serum biomarkers for hepatitis B virus: current status and future outlook. Nat Rev Gastroenterol Hepatol.

[71] Chigbu DGI, Loonawat R, Sehgal M, Patel D, Jain P. (2019). Hepatitis C virus infection: host–virus interaction and mechanisms of viral persistence. Cells.

[72] Shoraka S, Hosseinian SM, Hasibi A, Ghaemi A, Mohebbi SR. (2023). The role of hepatitis B virus genome variations in HBV-related HCC: effects on host signaling pathways. Front Microbiol.

[73] Suhail M, Sohrab SS, Kammal MA, Azhar EI. Role of hepatitis c virus in hepatocellular carcinoma and neurological disorders: an overview. Front Oncol. 12:913231. 10.3389/fonc.2022.913231 PMC9372299 35965577

[74] Chakladar J, Wong LM, Kuo SZ, Li WT, Yu MA, Chang EY (2020). The liver microbiome is implicated in cancer prognosis and modulated by alcohol and hepatitis B. Cancers (Basel).

[75] Zaki MYW, Fathi AM, Samir S, Eldafashi N, William KY, Nazmy MH (2022). Innate and adaptive immunopathogeneses in viral hepatitis; crucial determinants of hepatocellular carcinoma. Cancers (Basel).

[76] Aggarwal N, Kitano S, Puah GRU, Kittelman S, Hwang IY, Chang MW. (2023). Microbiome and human health: current understanding, engineering, and enabling technologies. Chem Rev.

[77] Spencer SP, Fragiadakis GK, Sonnenburg JL. (2019). Pursuing human-relevant gut microbiota-immune interactions. Immunity.

[78] Son G, Kremer M, Hines IN. (2010). Contribution of gut bacteria to liver pathobiology. Gastroenterol Res Pract.

[79] Chang AC, Parsonnet J. (2010). Role of bacteria in oncogenesis. Clin Microbiol Rev.

[80] Khanam A, Chua JV, Kottilil S. (2021). Immunopathology of chronic hepatitis B infection: role of innate and adaptive immune response in disease progression. Int J Mol Sci.

